# 4 Steps To My Future (4STMF): protocol for a universal school-based pilot and feasibility study of a CBT-based psychoeducational intervention to support psychological well-being amongst young adolescents in the Western Cape, South Africa

**DOI:** 10.1186/s40814-022-01035-x

**Published:** 2022-05-05

**Authors:** Bronwynè J. Coetzee, Maria E. Loades, Suzanne Human, Hermine Gericke, Helene Loxton, Gerrit Laning, Naomi Myburgh, Paul Stallard

**Affiliations:** 1 Department of Psychology, Stellenbosch University, Stellenbosch, South Africa; 2 Department of Psychology, University of Bath, Bath, UK; 3 Community Keepers, Non-Profit Company, Stellenbosch, South Africa; 4 Department of Health, University of Bath, Bath, UK

**Keywords:** Anxiety, Depression, Prevention, Universal, School-based, Mental health, CBT-based, Psychoeducational intervention, Pilot, South Africa

## Abstract

**Background:**

Mental health problems often emerge during middle childhood and adolescence. In South Africa, and in the context of high rates of poverty, violence, and adversity, many children are at a considerable risk for developing mental health problems. Access to and costs of mental health services preclude treatment for most. There is evidence that universal school-based prevention programmes are effective in well-resourced settings. However, little is known about the feasibility and acceptability of such programmes in low- and middle-income countries (LMICs), including South Africa.

**Methods:**

This is a feasibility pilot study of 4 Steps To My Future (4STMF), a Cognitive Behaviour Therapy (CBT) school-based programme for young adolescents in the Western Cape, South Africa. This eight-session intervention will be delivered to children in grade 5 (aged 10–13 years approximately) attending two public government-run schools in the Western Cape, South Africa. We aim to enrol approximately 224 children in grade 5. We will randomise which school receives the intervention first and the other will be a delayed intervention group. We will train individuals with a post-graduate degree in psychology to facilitate the programme. We will collect demographic data on participants as well as data on primary (feasibility measures) and secondary outcomes (mental health and well-being measures). We will collect data at baseline, post-intervention, and at 1-month follow-up.

**Discussion:**

This pilot study will provide data on the acceptability and feasibility of delivering a universal school-based prevention programme in South African schools. The study will provide preliminary data to inform the design of a full-scale randomised controlled trial (RCT) of a universal school-based mental health programme aimed at preventing mental health problems.

**Trial registration:**

This trial is registered with the Pan African Clinical Trial Registry (https://pactr.samrc.ac.za/TrialDisplay.aspx?TrialID=10881) database, with unique identification number for the registry: PACTR202004803366609. Registered on 24 April 2020.

## Background 

Globally, at least 1 in 5 children and adolescents experience mental health problems, and this number is likely to be even higher in low- and middle-income countries (LMICs), like South Africa [1], where vulnerable populations face multiple adversities [2]. In South Africa, the prevalence of anxiety disorder symptoms amongst children and adolescents is reported to be high, ranging from 22 to 25.6% amongst 7–13 years old, in the Western Cape Province [3]. Normative data from a number of studies conducted within the same context over the past decade (for example Burkhardt et al., 2003 [4], 2012 [5]; Muris et al., 2006 [6], 2008 [7]) consistently confirmed higher fear and anxiety levels in South African children, compared to their western counterparts. Thus, even before the COVID-19 pandemic, children and adolescents in South Africa were already particularly at risk of developing mental health problems because they are exposed to multiple risk factors such as violence, child maltreatment, living in households affected by HIV/AIDS, and poverty [1, 8–10]. Furthermore, the COVID-19 pandemic is having a profound effect on all aspects of society, including mental health [11]. Whilst disease containment measures (DCMs) have been implemented to help reduce the spread of the virus, such measures have had several unintended adverse consequences for children and young people (CYP). For example, learning was disrupted and social and emotional support was reduced [12–14].

Finding appropriate, cost-effective, and efficient ways to intervene is a key priority, given the impact of mental health problems both short and long term. In the short term, we know anxiety and depression impact on daily functioning, disrupt educational attendance and attainment, affect social relationships and interfere with normative development [10, 15–18]. In the long term, untreated depression is associated with an increased risk of subsequent depression, interpersonal difficulties, and suicide in adulthood [19, 20].

There is convincing evidence, predominantly from high-income countries (HICs), that psychological treatments, including Cognitive Behaviour Therapy (CBT), are effective in treating anxiety and depression [21–25]. CBT-based programmes for CYP with anxiety have been widely used in individual and group-based contexts [26]. There is emerging evidence of the effectiveness of CBT-based approaches in these populations in LMICs [27–35]. Paradoxically, in these countries, there is also a lack of trained clinicians, particularly in the most deprived areas, where the vulnerability factors for developing mental health problems are highest [1, 9, 10]. This lack of trained clinicians has led to interest in mental health prevention programmes, but to date, preventive interventions undertaken in LMICs are unfortunately lacking. A recent systematic review conducted by our team of universal school-based mental health programmes in LMICs identified 12 studies conducted in 11 different LMICs with children aged 8–19 years of age [36]. Whilst five studies reported improvement in depression, and five studies reported improvement in anxiety, overall, there were limited data on the outcomes of the studies, and only four provided explicit examples of how the interventions were developed or adapted for the local context. Also, none of the studies was conducted in South Africa, and none of the studies involved parents or caregivers in a direct way.

Whilst nearly 90% of all children live in LMICs, only 10% of randomised trials are undertaken in these countries, with almost all being psychopharmacological trials [1]. This highlighted the need to develop and evaluate mental health prevention programmes for children in LMICs with schools providing a promising context for their delivery [37].

### Formative work that informed the intervention development

The design and adaptation of effective preventive interventions require community ownership, cultural flexibility, and fit with the delivery context to maximise effectiveness, appropriate training, and support to deliver, and relevance and acceptability to stakeholders [1, 29]. The intervention presented in this protocol is based on extensive formative work by the investigators. Firstly, this intervention is based on expertise in the use of CBT, programme development and evaluation, and experience of delivering preventive interventions in South African schools. Indeed, previous research by the investigators has demonstrated that existing evidence-based CBT-based activities and programmes can successfully be adapted to be culturally sensitive and to fit within the South African context [29–34, 38, 39]. Secondly, the intervention is informed by a systematic review by the investigators referred to earlier [36]. The findings of the review demonstrated that universal school-based approaches hold promise for reducing symptoms of anxiety and depression amongst CYP and are better delivered in group format and when based on principles of CBT. Thirdly, the intervention is informed by formative qualitative interviews conducted by the investigators with CYP (grades 5–7), school mental health counsellors, parents/caregivers, and teachers. These interviews elicited participant perspectives on what a universal school-based mental health intervention in this setting should look like and who should be involved [40]. Lastly, to adapt the intervention in the context of COVID-19, the intervention is informed by formative follow-up qualitative interviews with CYP (grades 5–7), school mental health counsellors, parents/caregivers, and teachers. These interviews asked participants about the challenges surrounding COVID-19 and various disease containment measures [41].

This formative work culminated in the manualised, psychoeducational CBT-informed intervention, *4 Steps To My Future* (4STMF). The four core steps of the programme are based on the principles of CBT and are designed to enhance self-esteem, promote helpful thinking, develop emotional regulation, and empower goal-focused action. Each of the four core steps is designed to be delivered over two brief school lessons lasting for 20–25 min per lesson (approximately 3–4 hours in total). Each lesson includes whole group and individual tasks. Whilst small group tasks were included in the original 4STMF, we had to adapt these in the light of COVID-19 so as to maintain safe social distancing. Further details on the programme content and activities are available from the authors on request. Participating children will be asked to complete tasks in between lessons to apply the skills learned in daily life. Classroom posters and some tangible materials (such as worksheets and notebooks) will provide personal reminders of the skills learned at each step. An informational handout will be sent to parents/caregivers after each step to inform them of the key learning points, what was done in the lessons, and how they can support this at home.

### Feasibility trial objectives

The primary objective of this study is to determine the acceptability and feasibility of the 4STMF programme. We will use a mixed methods design utilising quantitative and qualitative methods. We will determine acceptability and feasibility through assessing consent and assent rates, as well as session and programme completion rates. Fidelity checklists will be completed by the programme facilitators and independent observers for each lesson. These fidelity checks will elicit how confident, prepared, and enthusiastic facilitators appeared whilst delivering the programme, as well as how well they managed the classroom. Furthermore, the fidelity checklists will elicit how long it took to deliver a lesson, whether an activity was delivered or changed, and how confident, prepared, and enthusiastic the facilitators appeared during the delivery of that lesson. Furthermore, teachers will be asked to complete an evaluation form once the programme has been facilitated. Qualitative, semi-structured exit focus groups will be conducted with CYP following intervention delivery. We will determine the acceptability of our assessment measures (completion rates) and will explore pre- and post-intervention changes to determine sample size (power) for a future randomised controlled trial (RCT).

## Methods/design

**Fig. 1:**
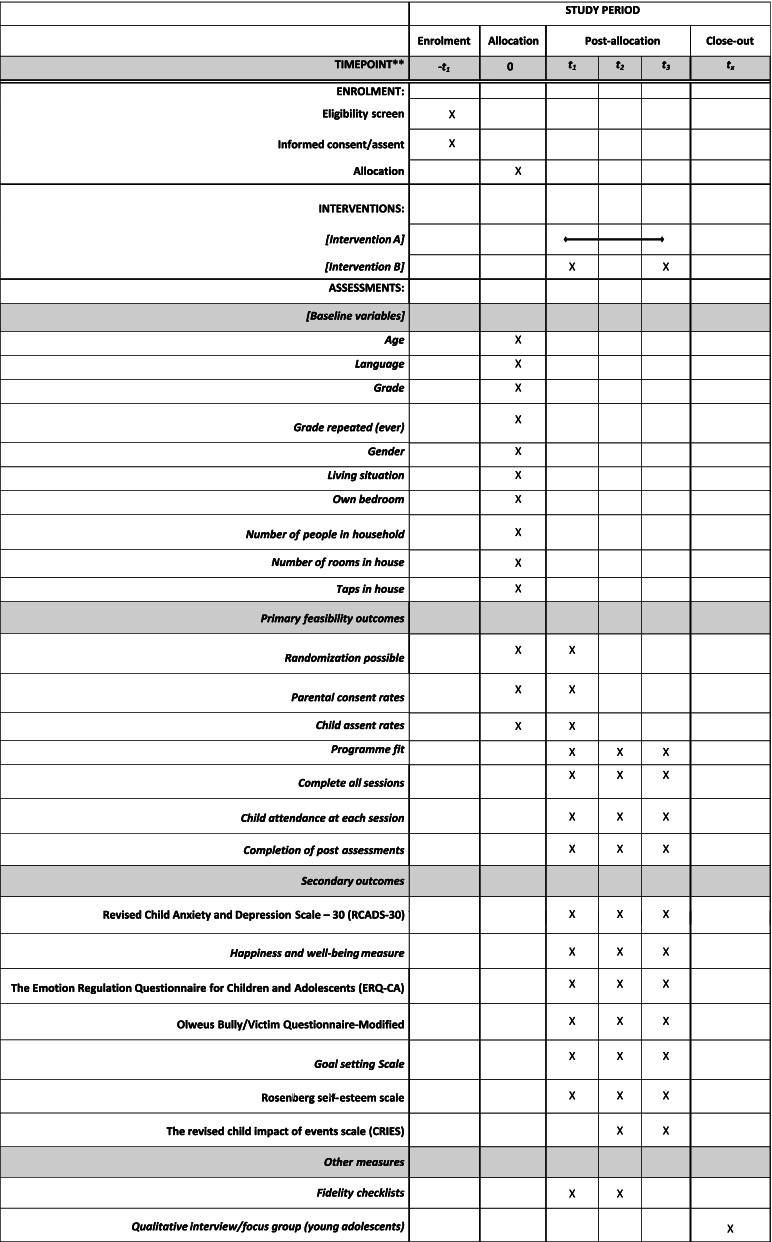
Standard Protocol Items: Recommendations for Clinical Trials (SPIRIT) figure—schedule of data collection

This paper reports on the protocol for the feasibility of a pilot intervention of the 4STMF programme in accordance with the Standard Protocol Items: Recommendations for Interventional Trials (SPIRIT) checklist (see Fig. 1).

### Trial design

**Fig. 2:**
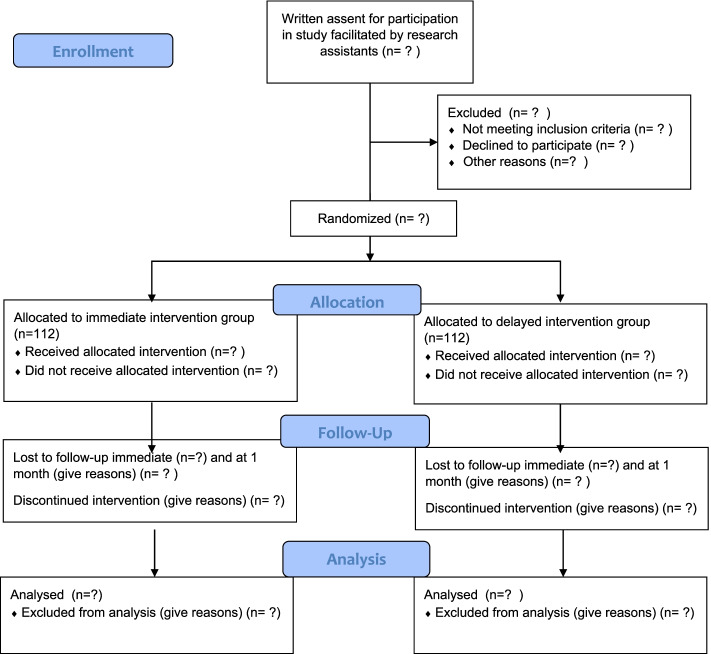
Consort diagram of the study design for the 4STMF programme

We will conduct a two-arm, randomised, feasibility pilot trial comparing immediate intervention group (IIG) delivery of 4STMF to a delayed intervention group (DIG). The consort diagram of the study design is shown in Fig. 2.

### Study setting

We will recruit participants from two public primary schools in the Western Cape province of South Africa and in collaboration with a non-governmental organisation (NGO). The NGO operates onsite within schools in the Western Cape to improve the social and emotional well-being of children and promote supportive school communities. The two schools were randomly chosen (using computer randomisation) from a list of schools (*N* = 21) within which the NGO operates. Both schools have a staff to learner ratio of approximately 40:1, and both schools form part of South Africa’s National School Nutrition Programme [42]. Schools are eligible for funding for this nutrition programme when most of the children come from low socio-economic status families. In total, each school has approximately 30–34 teachers and 900–1000 pupils.

### Characteristics of participants

Participants will be in grade 5 at participating schools (aged ~ 10–13 years). The intervention will be universally delivered, i.e. delivered to all participating children regardless of symptomatology or risk. Given the restrictions posed on this research by COVID-19 and given changes to school scheduling, we pragmatically decided to target the intervention at one grade, grade 5, only. There are approximately 20 eligible children per class with 6 and 8 classes at our target schools respectively. As such, whilst there are approximately 280 children eligible to take part in grade 5 across these 2 schools, we expect a sample of approximately 224 children to take part in this study which accounts for a 20% non-participation rate. We will calculate consent and retention rates at immediate post-intervention as well as 1-month follow-up. As per communication received from the Western Cape Education Department (WCED), no research may be conducted during the fourth term (October–December 2021) as schools are preparing and finalising syllabi for examinations. This could then mean that a 1-month follow-up assessment might not be possible with the children in the DIG.

### Ethical review and consent

Ethical approval has been obtained from Stellenbosch University’s Research Ethics Committee: Social Behavioural and Education Research (project number: 9183), and reciprocity has been received from the Psychology Research Ethics Committee (reference number: 19-073) at the University of Bath. The WCED approved of the study being conducted in the two schools (reference: 20200214-4483).

All grade 5 children in both schools will be informed about the intervention and will be given a project information sheet to take home. The information sheet will include an opt-out consent form to be returned if parents/caregivers do not wish their child to be part of the intervention. In addition, children will also be asked to complete assent forms prior to completing baseline assessments. Children whose parents do not agree to participate or who themselves do not provide assent will be supervised in a separate classroom. Children will not be required to provide reasons for not taking part. Once consent and assent have been received, post-graduate psychology students will administer the baseline battery of measures to children one class at a time. The programme facilitators will be on standby should children require individual assistance with the completion of the measures.

### Procedures

Assessments will be completed at baseline, post-intervention, and at 1-month follow-up. Measures will be completed in school, over two or three lessons. Researchers with a post-graduate degree in Psychology will read assessment items out loud as children individually respond to each question in their assessment booklet. Participants will have the option to complete forms in either English or Afrikaans. We will provide children with a thank you gift in the form of a 4STMF wrist band after completion of the intervention sessions, and the completed assessment battery.

The language of the programme delivery will be in the predominant language of each class, either English or Afrikaans. Programme facilitators will be trained to deliver the programme fluently in both languages. Class teachers will be encouraged to attend the lessons delivered and may be involved in disciplinary processes if necessary, during the lessons, but will not be expected to deliver the programme material.

Following completion of the 4 Steps To My Future (8 lessons) programme, children will complete the assessment battery, and again at a 1-month follow-up. On completion of intervention delivery, a subgroup of participants stratified to include both male and female participants will be invited to take part in focus group discussions to share their experiences of the programme.

### Outcome measures

**Fig. 3:**
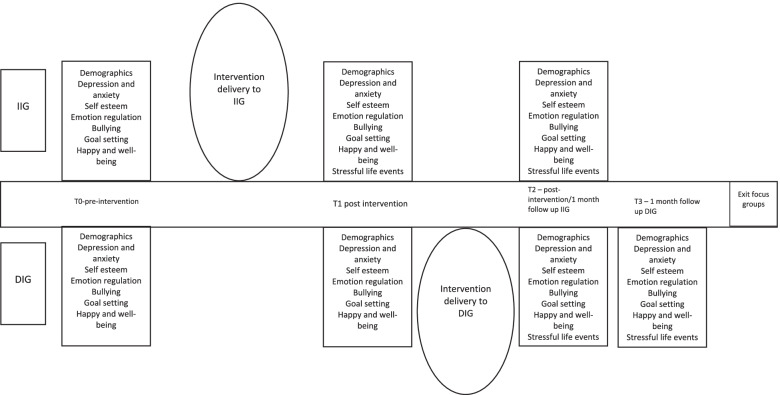
Overview of the project timeline. IIG immediate intervention group, DIG delayed intervention group

All participants will provide demographic information at baseline (see Fig. 1). The schedule of data collection is shown in Fig. 3.

### Feasibility

To determine whether it is feasible to deliver the intervention and assessment measures, we will collect the following feasibility and acceptability outcomes:

1. Rates of parental opt-out: How many parents/caregivers refused consent for their child to take part in the programme? For this outcome, we will count the number of consent forms returned from parents. As mentioned, we will use parental opt-out. As such, parents who sign and return the consent form will do so indicating that they do not want their child to take part in the study.
2. Rates of child assent: How many children declined to take part in the programme? For this outcome, we will count the number of assent forms handed out and then the number of forms returned by learners invited at baseline. We will provide each child in grade 5 with an assent form. We will subtract the number handed out from the number of forms returned.
3. Rates of assessment completion: How many participating children completed assessments at baseline, post-intervention, and 1-month follow-up? For this outcome, we will count the number of children who completed assessments at each time point. We will subtract the number of completed measures from the number who provided consent at baseline to determine how many assessments were not completed at each time point.
4. Programme completion: How many groups received all 8 sessions of 4 Steps To My Future? For this outcome, observers will document session delivery to each group/class of learners. Observers will capture whether a session was delivered in full or not.
5. Session attendance: How many children attended each session of the programme? For this outcome, we will count the number of learners present in class for each session delivery. We will then obtain class attendance records from the class teacher to document which learners (who consented to take part) were present or absent.
6. Programme fidelity: How many programme sessions were delivered fully, as intended. For this outcome, observers will capture on observation templates whether a session was delivered fully as intended or not. The observer will be instructed to note what circumstances interfered with session delivery as intended.

### Acceptability

Acceptability will be determined through exit focus groups with a sample of young people who participated in the programme. Focus groups will be arranged in a safe place in a private classroom or office on the school premises. All participating children will be invited to take part in the exit focus groups. Following parental/caregiver consent as well as assent from children themselves, approximately three children per class will be randomly selected and we will aim to include both male and female participants. The exit focus groups will not be conducted by the programme facilitators, but by independent, trained post-graduate psychology students or members of the research team. We intend to conduct at least 1 focus group in each school with up to 6 children in each focus group. Focus groups will be guided by a script which will assess a range of domains including acceptability, understanding, relevance, and helpfulness. Focus group interviews will be audio-recorded with permission from the children.

### Psychological well-being

We will undertake an exploratory analysis of our psychological measures. The purpose will be to (i) inform decision-making about which will be our primary outcome in a subsequent trial and (ii) to inform the power calculation for a subsequent RCT. The following standardised psychological measures will be completed at each assessment.

#### Symptoms of depression and anxiety

We will use the Revised Child Anxiety and Depression Scale-30 (RCADS-30) [43] to measure symptoms of depression and anxiety. The 30-item measure asks participants to rate their responses on a 4-point Likert scale from ‘never’ to ‘often’. The measure has good psychometric properties, and amongst a South African sample of adolescents, the 10-item depression subscale showed good internal consistency (alpha coefficient = 0.86) [44].

#### Happiness and well-being

We will use the happiness and well-being measure developed as part of the PACES trial [45]. The 7-item, visual analogue scale measures happiness about the school, appearance, family, friends, home, health, and life in general.

#### Emotion regulation

We will use the 10-item Emotion Regulation Questionnaire for Children and Adolescents (ERQ-CA [46];) to measure emotion regulation strategies of cognitive reappraisal (6 items) and expressive suppression (4 items). The measure has sound internal consistency and shows stability over 12 months.

#### Bullying

We will use the 2-item Olweus Bully/Victim Questionnaire-Modified [47]. Response options are given on a 5-point rating scale (0 not at all, 1 = once or twice, 2 = two or three times a month, 3 = about once a week, 4 = several times a week). The measure has 9 items; however, we will only use two. We use the two items used as part of the PACES trial [45] in which children are asked about how often they have been bullied, and how often they have taken part in bullying other children.

#### Self-esteem

We will use the 10-item Rosenberg self-esteem scale [48]. Rosenberg’s Self-Esteem Scale is the standard measure of self-esteem in psychological research. The scale provides a short, straightforward, and convenient method for measuring global self-esteem. Items are measured on a 4-point rating scale from 1 (strongly agree) to 4 (strongly disagree).

#### Goal setting

We will use the 5-item goal setting scale [49]. Reliability analyses yielded an alpha of 0.68 for this scale.

#### Stressful life events in the past 7 days

The revised child impact of events scale (CRIES) (at post-intervention only) [50]. This measure contains a screening question at the beginning to assess for eligibility for completing this measure. As such, not all children will necessarily be eligible to complete this measure in full.

#### Translation of measures

Each of these measures is available freely in the public domain for research purposes. These measures as well as the programme will be available in both English and Afrikaans. All the measures are available in English. Measures not available in Afrikaans were professionally translated into Afrikaans and professionally and independently back translated into English. The translations were checked for accuracy by first language Afrikaans-speaking members of the research team (SH, HG, HL, NM).

### Randomisation and blinding

Two schools were randomly selected from a list of 21 schools within which the NGO operates. These two schools were then randomised to either immediate or delayed delivery of 4STMF. By the nature of the intervention, neither the participants nor the interventionists were blind to arm.

### Intervention

The intervention will be delivered during the Life Orientation (LO) or Personal and Social Wellbeing (PSW) lessons, or at a time deemed most suitable by the respective school principals and teachers. The LO and PSW lessons are part of the South African national education curriculum and are aimed at providing children with basic skills and knowledge regarding health, society, rights and responsibilities, physical education, and preparation for the world of work.

Both the immediate intervention group as well as the delayed intervention group will receive the programme in a face-to-face whole class delivery format.

The intervention is informed by CBT and throughout the 8 sessions teaches skills in four main areas. Table 1 provides an overview of the components of the programme. Firstly, participants are encouraged to develop their self-esteem through identifying their personal strengths, accepting who they are, and being kind to themselves and others. The second introduces children to their cognitions and the importance of developing “go” thinking (positive, enabling, and balanced). Thirdly, children are encouraged to attend to how they feel and to positively manage strong unpleasant emotions. Finally, children are encouraged to identify their future goals, to break these down into steps, and to learn to problem solve to address issues that might impede their attainment.

**Table 1:** Components of the 4STMF intervention package

Theoretical framework (intervention)	Cognitive behavioural therapy (CBT) based with a focus on psychoeducation, resilience, skills-building, and well-being
**Interventionists/delivery agents**	• Registered counsellors/masters-level psychology students with 1–2 years of research experience and who attended training on the intervention package; masters-level psychology students with 1–2 years of research experience who will act as observers and conduct fidelity checks
**Structure of intervention package**	• Eight × 20–25-min sessions, delivered over 4 to 8 weeks (delivered either weekly or twice weekly depending on fit with school)
**Structure of sessions**	• The programme is split into 4 steps — and each step contains 2 lessons. Participants in the immediate intervention group (IIG) and the delayed intervention group (DIG) receive the intervention
**Step 1 lesson 1**	• Introduction to 4STMF (5 min)
	• Self-esteem and respect (10 min)
	• Remember your strengths (6 min)
	• Home task saying my strengths out loud each morning (4 min)
**Step 1 lesson 2**	• Recap of the previous session (5 min)
	• Accept who you are (10 min)
	• Be kind to yourself and others (6 min)
	• Home task spreading kindness (4 min)
**Step 2 lesson 3**	• Recap step 1, value who you are (10 min)
	• The way you think (10 min)
	• Home task recognising my STOP and GO thoughts (5 min)
**Step 2 lesson 4**	• Recap of the previous session (5 min)
	• GO thinking (10 min)
	• Home task and identification of GO thoughts (10 min)
**Step 3 lesson 5**	• Recap step 2, thinking that I can (5 min)
	• Recognise how you feel (15 min)
	• Home task how I feel (5 min)
**Step 3 lesson 6**	• Recap of the previous session (5 min)
	• Helping yourself to feel better (15 min)
	• Home task and practice (5 min)
**Step 4 lesson 7**	• Recap step 3, and practice relaxation (5 min)
	• My goals (15 min)
	• Home task my goals (5 min)
**Step 4 lesson 8**	• Recap of the previous session (5 min)
	• Problem solving (15 min)
	• Recap and ending (5 min)
**4STMF training**	
** Structure and format**	• Once-off × 6–7 h of facilitator training with lead implementer; either online or in person. The training takes place over 2 days.
** Training content**	• Training content includes an overview of core components of CBT, an overview of programme development, orientation to session content, and role playing of lessons; orientation to measures and fidelity checks
** Characteristics of supervisor(s)**	• Counselling psychologist registered with the HPCSA with more than 5 years counselling experience and experience in CBT-based interventions; post-doctoral researcher with more than 5 years of CBT intervention delivery experience
** Structure of supervision and debriefing**	• Counselling psychologist and post-doctoral researcher meet with intervention implementers in person or online/telephonic, once after the completion of each step of the programme (so once after two sessions have been delivered). As such 4 × 1-h supervision debriefing sessions
	• An hour session of debriefing/supervision which entails reflections on the delivery and adherence and challenges with delivery and content
	• Research group debriefing (core research team and implementers), once-off after the delivery of 8 sessions at each of the schools

The intervention will be delivered by 2 trained facilitators. It is designed to be active and engaging and uses a mix of whole group exercises, individual exercises, and different formats (speech, writing, reading of stories, role play, hand gestures, and visual posters). After each session, children will be asked to undertake a home assignment to transfer the skills learned in the classroom to their everyday life.

#### Intervention facilitators’ training and supervision

Intervention facilitators will have at least an undergraduate degree in psychology or social sciences and will attend a 2-day training workshop covering the theoretical underpinnings of CBT, specific training on the format, content and delivery of the programme, and procedures for monitoring and evaluation. Both facilitators will be assisted by post-graduate psychology students and/or school mental health counsellors from the NGO, who will act as observers during the facilitation of the lessons. All intervention facilitators will have attended a 2-day training workshop covering the theoretical underpinnings of CBT, specific training on the format, content and delivery of the programme, and procedures for monitoring and evaluation. The training is structured to be a combination of didactic instruction and skills development through role play and reflective discussions. For the purposes of fidelity to the programme and to discuss delivery issues, NM (who has expertise in teaching and in delivering CBT-based mental health interventions to young children in South Africa) and/or HL (who is a counselling psychologist and lecturer with expertise in the development, implementation, and evaluation of CBT-based anxiety interventions for youth in South African) and/or BC (a lecturer and researcher in child mental health and study principal investigator (PI)) and/or ML (who is a clinical psychologist and lecturer with extensive experience of delivering CBT to children and young people) will conduct 1 h of supervision with the lead interventionist and co-facilitator after each step of the programme has been delivered. As such, the programme facilitators will receive 4 h of supervision from an experienced CBT practitioner during the course of the programme.

#### Data analysis

Descriptive statistics will summarise our feasibility outcomes and demographic data. Quantitative data will be analysed using SPSS and other statistical software, as appropriate. Participant characteristics will contain both continuous and categorical variables. We will use means and standard deviations to summarise data collected on the continuous variables and frequencies and percentages to summarise data collected on the categorical variables. An exploratory analysis of the psychological outcomes will be undertaken. Analysis will involve pre- and post-comparisons within and between groups exploring baseline, post-intervention, and 1-month follow-up data.

Qualitative data will be transcribed verbatim and will be uploaded into ATLAS.ti 9 to be analysed deductively and/or inductively using both content analysis and thematic analysis [51]. Qualitative data will be focus group data collected from learners on completion of intervention delivery at both schools.

The findings obtained from our qualitative formative work were used to inform the development of the intervention material. Similarly, we will integrate both quantitative and qualitative data obtained from this feasibility and acceptability pilot study in our reporting of the main study outcomes. We will do so by providing descriptive data on our feasibility outcomes and complementing these numerical data with qualitative data obtained from our exit interviews. These exit interviews may then help us to understand qualitatively, for example, why the intended number of sessions was not completed.

## Discussion

There is an urgent need for mental health programmes to focus on CYP in resource-limited settings. This study will be the first to explore the acceptability and feasibility of a universal CBT-based, school-based mental health intervention delivered to CYP in two primary schools in the Western Cape, South Africa. It is designed to assess questions of acceptability and feasibility in order to inform a full-scale RCT. Both quantitative and qualitative data will be used to assess acceptability and feasibility outcomes. The intervention is designed to be delivered by individuals with a psychology background within school settings that have available psychosocial support services and, if subsequently proven to be effective, could be readily delivered at scale. This protocol was originally conceptualised before the COVID-19 pandemic, and at the time of this writing, the Western Cape has emerged from a 3rd wave of the pandemic. Depending on COVID-19, this protocol may need to be amended in order to accommodate any changes (e.g. school closures, social distancing rules) that may impact on the successful completion of this study. We have written this protocol for a pilot study with a particular context and setting in mind. It is important to consider that any future adaptation of this intervention within other contexts will need to be carefully considered and these adaptations may have implications on estimated treatment effects.

## Data Availability

The datasets used and/or analysed during the current study will be made available from the corresponding author on reasonable request.

## Abbreviations

CBT: Cognitive Behaviour Therapy
COVID-19: Coronavirus disease 2019
CYP: Children and young people
DBE: Department of Basic Education
DCMs: Disease containment measures
DIG: Delayed intervention group
DRD: Division for Research Development
ERQ-CA: Emotion Regulation Questionnaire for Children and Adolescents
HIC: High income countries
IIG: Immediate intervention group
LMICs: Low- and middle-income countries
LO: Life Orientation
PSW: Personal and Social Wellbeing
RCADS-30: Revised Child Anxiety and Depression Scale-30
REC: SBER: Research Ethics Committee: Social, Behavioural and Education Research
RCT: Randomised controlled trial
SPSS: Statistical Package for the Social Sciences
WCED: Western Cape Education Department
4STMF: 4 Steps To My Future
